# Cultural considerations in the assessment of sensitivity in low-income caregivers in Ethiopia

**DOI:** 10.3389/fpsyg.2023.1163773

**Published:** 2023-04-26

**Authors:** Maria Spinelli, Emanuele Preti, Temsegen Tadele Kassa, Moges Ayele Asale, Mulat Asnake Goshu, Tigist Wuhib Tsega, Abebaw Minaye Gezie, Mirco Fasolo, Judi Mesman

**Affiliations:** ^1^Department of Neuroscience, Imaging and Clinical Sciences, University G. d'Annunzio, Chieti, Italy; ^2^Department of Psychology, University of Milano-Bicocca, Milan, Italy; ^3^Integrated Family Services Organization (IFSO), Addis Ababa, Ethiopia; ^4^School of Psychology, College of Education and Behavioral Studies, Addis Ababa University, Addis Ababa, Ethiopia; ^5^Faculty of Social and Behavioural Sciences, Institute of Education and Child Studies, Leiden University, Leiden, Netherlands

**Keywords:** sensitivity, low-income families, discipline, Ethiopia, caregiver–child interaction

## Abstract

**Background:**

Caregiver sensitivity is associated with positive child outcomes, and improving sensitivity is often the aim of parenting-directed interventions. However, sensitivity was conceptualized in Western cultures, and its application in populations with different backgrounds is still limited.

**Objective:**

This study aimed to foster a contextualized cultural understanding of the meaning and nature of sensitivity by assessing the possibility of evaluating sensitivity in a low-income population living in Ethiopia and describing the nature of (in)sensitive parenting. Moreover, the associations between sensitivity and discipline, the quality of the environment, and individual characteristics were explored.

**Methods:**

Parental sensitivity was coded on naturalistic video-recorded observations of free interactions between 25 female primary caregivers and their children. Caregivers completed questionnaires on discipline strategies and the level of satisfaction with the environment (access to basic needs, quality of house condition, community and family support, quality of learning opportunities, and working conditions).

**Results:**

The assessment of sensitivity in this population was possible, with caregivers showing the full range of sensitivity levels. A description of manifestations of sensitivity in this population is provided. A K-means cluster analysis evidenced that high sensitivity was associated with high satisfaction regarding housing conditions and family environment. No association between sensitivity and discipline emerged.

**Conclusion:**

The findings show the feasibility of assessing sensitivity in this sample. The descriptions of observed behaviors contribute to understanding culturally specific aspects of sensitivity to consider when assessing sensitivity in similar populations. The study provides considerations and guidelines to inform the structure of culturally-based interventions to promote sensitive parenting in similar cultural and socioeconomic situations.

## Introduction

Caregiver sensitivity is a construct first defined and operationalized by Mary Ainsworth, within the framework of attachment theory. It refers to a caregiver’s ability to notice infant signals, interpret them correctly, and respond promptly and appropriately by adapting behaviors to the infant’s needs (Ainsworth et al., 1974; Bowlby, 1988). Based on this definition, the evaluation of caregiver sensitivity has been widely applied in research and clinical settings using the appropriately designed Ainsworth’s sensitivity scale (Ainsworth et al., 1974). Sensitivity is hypothesized to be a universal aspect of parenting, and because the appropriateness of the sensitive response is evaluated based on the child’s feedback and not on the type of caregiver’s behavior, it is supposed to be present across cultures and contexts (Mesman et al., 2018b; Mesman, 2020b).

Given the evolutionary advantage of being reared by a proximal and responsive caregiver prone to appropriately take care of children’s needs, sensitivity is related to positive child development (Ainsworth et al., 1974; Mesman et al., 2012, 2016b; Chapin, 2018). For this reason, promoting sensitivity is a focus of many interventions directed to parents: improvements in sensitivity lead to better child outcomes in several domains, i.e., attachment, emotional development, and behavioral regulation (Morawska et al., 2019; Cooke et al., 2022; van IJzendoorn et al., 2023).

Most interventions focused on sensitivity, and its manifestations are based on the notion of the sensitive caregiver as derived from Western-based observational studies. However, recent studies explored the expressions of sensitivity in different cultures and found culture-specific patterns and manifestations (Mesman et al., 2018b; Mesman, 2020b). Understanding and describing how caregivers express their sensitivity within their specific cultural context and living condition is essential, not only to test the universality of sensitivity but also to examine whether sensitivity could be a meaningful caregiving construct to target in culturally sensitive interventions aimed at promoting positive parenting and child development.

To contribute to this new field of culture-specific studies, the present study evaluated caregiver sensitivity during observations of naturalistic interactions with Ainsworth’s scale and assessed its application and reliability in a group of low-income women and their children living in poor suburban areas of Addis Ababa, Ethiopia. Descriptions of manifestations of sensitivity are provided to help clinicians apply this assessment in similar populations. In addition, the associations between sensitivity and relevant caregiver’s characteristics were tested. This study is the first to assess sensitivity and describe typical (in)sensitive caregiving behaviors in this population that shares socioeconomic and cultural factors with many families living in poor sub-urban areas in East Africa. These populations are often considered at risk for adverse child development outcomes and are therefore the target of many NGOs’ and local services’ supportive and preventive intervention programs. The findings would yield information that care professionals can use to structure more culturally-defined interventions aimed at caregivers of young children (Bornstein, 2019).

### Parenting in Ethiopia

Ethiopia, the second most populous country in Africa, is the home of an estimated 105 million people, with the largest urban population living in the capital city, Addis Ababa (CSA, 2013). Like other African countries, Ethiopia seems to have a predominantly collectivist culture (Hofstede, 1983), or more precisely a vertical collectivist one, characterized by strong in-group cohesion and a focus on complying with authorities and respect for hierarchy (Triandis and Gelfand, 1998). Indeed, collectivist values, such as togetherness, support, interdependence, compliance and agreement among group members, as well as hierarchical arrangements and obedience, are prominent in the Ethiopian context (Gota, 2012) and are promoted within the family (Ringness and Gander, 1974; Teferra et al., 1996; Poluha, 2007).

Only a few studies, primarily unpublished dissertations, have examined the manifestations of parenting in Ethiopia. In these studies, researchers often used questionnaires to examine parenting styles reported by offspring, mainly high school and university students, with mixed results. While two studies showed the presence of supportive and collaborative parenting styles (Gota, 2012; Mekonnen, 2017), most studies described a different pattern. In one of the first published studies on the topic, students from rural Ethiopian areas when describing their parents’ child-rearing behaviors revealed the use of a harsh authoritarian style and a demand for unquestioned obedience to rules (Ringness and Gander, 1974). More recent studies confirmed these findings, revealing that the most frequently reported parenting style in rural Ethiopian areas is formal, authoritarian, and restrictive parenting that does not encourage independence or initiative in the young and tends to use more punitive and harsh discipline (Abraham, 1996). High school adolescents living in the urban area of Addis Ababa reported a similar predominance of an authoritarian parenting style characterized by clear rules and consequences, a lack of shared fun activities, unquestioning obedience, and close monitoring (Cherie and Berhanie, 2015). Interestingly, most participants described their parents also as willing to provide advice and reported high closeness between family members (Cherie and Berhanie, 2015).

The reason why Ethiopian parents appear to prefer the authoritarian parenting style was explored by a qualitative study based on interviews with parents living in very poor economic conditions in the suburban setting of Addis Ababa (Gelan, 2016). This study showed that parents believe that the frequent use of supervision, punishment, and control increases children’s respect for cultural and familiar values, facilitates harmony between family and community, and improves children’s ability to play adult roles as early as possible, and children’s opportunities to achieve educational and career success (Gelan, 2016). Consistent with this idea, quantitative studies reported that the more Ethiopian adolescents experienced an authoritarian parenting style and felt supervised and monitored, the less they showed risky sexual behaviors (Cherie and Berhanie, 2015) and the more they achieved higher academic scores (Tilahun, 2002).

Although these studies provide an important contribution to understanding general parenting in the Ethiopian context, they also have several limitations leaving many gaps in the literature. The present study aimed to fill these gaps.

First, all these studies, except one, used retrospective self-reports administered to adolescents or adults, mainly well-educated students. Thus, the studies do not capture parenting in the general population of Ethiopia, which often lives below the absolute poverty line (Ephrem, 2019). This is especially true in rural areas and in the sub-urban areas of big metropolitan cities, such as Addis Ababa (EPHI and ICF, 2019), where families live in poor housing conditions and shanty settlements, such as shacks, with only one small room shared by many family members (Meharie, 2009). Research on economic deprivation and parenting generally shows that poverty and stress associated with its pressures can undermine parents’ ability to be supportive caregivers, with potentially negative consequences for children’s development (Cooper et al., 2009; Mesman et al., 2012; Hoff-Ginsberg and Laursen, 2019; Dawson et al., 2020). However, studies that examined sensitivity in populations with very scarce economic resources reported mixed findings (Mesman et al., 2018a; Alsarhi et al., 2020; Asanjarani et al., 2020; Fourment et al., 2020). Exploring the levels and nature of parenting in Ethiopian families living in poverty is necessary to understand if there is variation within this population that might provide insights into sources of resilience that can be informative for interventions aiming to support parents and children (Hoff-Ginsberg and Laursen, 2019).

A further limit is the use of questionnaires that restricts the coverage of more subtle and complex aspects of parenting, such as sensitivity, that require (video) observation. Thus, the application of those findings for the structure of preventive and supporting interventions is limited. Consequently, most clinicians and social workers had to base their work on manuals and guidelines conceptualized for Western caregivers.

The lack of observational studies in Ethiopia is likely due to the difficulties of recruiting families for this type of research. Ethiopia is a large country with a wide variety of living conditions, from big metropolitan to rural areas. Moreover, its population is generally unfamiliar with scientific research that requires video observations, and the realities of daily life can put practical constraints on data collection (Mesman et al., 2016a, 2018b). Observing various spontaneous caregiving situations in the home environment rather than laboratory and structured assessments can be an excellent strategy to increase ecological validity. The description of spontaneous caregiving behaviors through the lens of sensitivity is relevant to provide insights into the nature and quality of interactions between caregivers and young children in Ethiopia. It can contribute to a fundamental guideline for health workers and clinical psychologists working with populations with similar socioeconomic conditions and cultural backgrounds to promote and support family wellbeing and child development (Bornstein, 2012; Mesman, 2020a).

### The present study

The present study, applying a mixed method design, aimed to gain more knowledge on the quality of parenting in Ethiopian families living in high poverty to inform culturally based interventions and preventive programs. Because this cultural background and socioeconomic condition are pervasive in sub-urban areas in Central Africa, the achieved knowledge could eventually be extended.

The current study’s first aim (Aim 1) was to explore if caregiver sensitivity could be assessed using Ainsworth’s scale Ainsworth et al. (1974) and whether different sensitivity levels were present in low-income families living in suburban areas of Ethiopia. We video-recorded spontaneous caregiver–child interactions in the home setting, and videos were coded following the original scale to assess caregiver sensitivity. Based on previous studies on sensitivity in different cultures, we expected to be able to apply the scale and find a full range of sensitive behaviors.

The second aim (Aim 2) was to describe manifestations of (in)sensitive parenting to contribute to the understanding of potential culturally specific aspects of sensitivity. The characteristics of caregiver–child behaviors and the type of activities conducted were discussed to provide examples of sensitive and insensitive behaviors specific to this population. Consistent with the recent literature on parenting in different cultures, we expected to find interactive behaviors specific to this cultural background and socioeconomic conditions (i.e., less play with toys, more culturally specific activities such as preparing typical food) but can be observed to be conducted in a sensitive or insensitive way.

The third aim (Aim 3) was to study the associations between sensitivity and other caregiver characteristics relevant to foster a contextualized cultural understanding of the meaning and nature of sensitivity. To do so, we examined the association between sensitivity and family socioeconomic characteristics, caregivers’ discipline behaviors, and perception of the quality of the environment. Because previous studies have shown that harsh parenting, characterized by less sensitivity and highly punitive behaviors, is often more common in caregivers living in difficult socioeconomic conditions, some studies conducted in Ethiopia found a prevalence of authoritarian parenting style associated with punitive behaviors, and we expected to find low sensitivity to be associated with low-quality socioeconomic and environmental living conditions and with more punitive discipline behaviors.

## Materials and methods

### Participants

A total of 25 principal female caregiver–child dyads participated (see Table 1). In 20 dyads, they were mostly the child’s mother, three were the aunt, and two were the grandmothers. In all cases, the principal caregiver took care of the infant from birth (in three cases, the mothers disappeared after delivery, and in two cases, the mother was dead) and will, from now on, be labeled as the caregiver in this study.

**Table 1:** Participants characteristics.

	*M* (*SD*)	*N* (Percentage)	Range
Child age (years)	4.10 (2.87)		5 months – 11 years
Gender (female)		16 (71%)	
Only child		9 (36%)	
Father alive		18 (72%)	
Co-residing family members (N)	5 (2.23)		2–10
One room house (N)		17 (68%)	1–2
Unemployed caregivers		10 (40%)	
Caregiver education (years)	4.5 (3.88)		0–10
Caregiver no school attending (N)		9 (36%)	

All the families lived in economically disadvantaged conditions, in a shack with one or a maximum of two rooms, with sheet metal roofs and earth floors, no bathroom, no running water, and only rare access to electricity.

Participants were part of the Child Sponsorship Project of Il Sole NGO and IFSO.^1^ The program was designed to address economic and psychosocial difficulties. Many children are exposed to the sub-urban area of Addis Ababa. Families living in very poor economic conditions, with only low irregular salaries gained with daily labor activities, often not enough to provide basic needs, such as food and clothes, receive a monthly financial contribution to cover the cost of school materials, medical, and other related essential expenses for the child. Financial help for emergency situations (i.e., house restoration, illnesses) is also provided. NGO’s local counselors and social workers proposed participation to all caregivers of children younger than 12 years without severe health issues that could compromise their interactive abilities. All contacted families consented to participation.

### Procedure

Participants were explained that the NGO was conducting a study, in collaboration with the University, on children and caregivers living in Addis Ababa with attention to caregiver–child interaction and related caregiving variables. The evaluation of the quality of caregivers’ interactive behaviors was not mentioned. Participants had the right to withdraw from the study at any stage if they wished to do so. Data were collected during a home visit organized at a day and time decided by the family. All the legally authorized caregivers signed an appropriate written consensus form including basic information about the study (i.e., the aim of questionnaires, video registration, privacy, etc.). The NGO’s staff read the consensus form to illiterate caregivers to collect their agreement. The video recordings were conducted by local university students, who were introduced to families by the NGO’s counselors. For these families, the home visit of NGO counselors is customary, and they feel comfortable in their presence. After some minutes of informal chatting to familiarize themselves with the researchers, caregivers were asked to interact with the child as they would normally do. They were free to choose what to do and to be filmed inside or outside the house. When possible, no other people were present in the house. In 87.5% of the videotaped interactions, the caregiver and the child were alone with the researchers; in the other cases, mainly brothers and sisters were present.

After the caregiver–child interaction, the caregivers completed the questionnaires. The questionnaires were translated with back-translation from English to the local language (Aramaic). Because most caregivers were illiterates, the researchers read the questions to the caregivers and noted the answers. To facilitate comprehension, visual representations of the Likert scales were used.

An ID was assigned to each caregiver–child dyad to protect participants’ privacy, and personal details were removed from the research material.

The study was approved by the ethical committee of the department [name omitted for blind review] and was conducted according to American Psychological Association guidelines in accordance with the, 2013 Helsinki Declaration.

### Instruments

#### Video coding of caregiver sensitivity

All video materials (length range 8–16 min) were transcribed in detail, and the caregiver’s and child’s speaking were translated into English by local researchers to facilitate the coding of sensitivity by the first and last authors. Caregiver sensitivity was coded using the Ainsworth Sensitivity versus Insensitivity scale (Ainsworth et al., 1974). The Ainsworth sensitivity scale ranges from *1 (highly insensitive)* to *9 (highly sensitive)* and highlights the extent to which a caregiver notices the infant’s signals and adapts her or his behavior accordingly to meet the infant’s needs. Coding was carried out by the first and the last author, and both were trained observers, the latter with extensive experience with the Ainsworth sensitivity scale in multiple cultural contexts. The first coder coded all the videos, and both authors coded 70% (*n* = 18) of the videos. Intercoder reliability (intraclass correlation), calculated on 11 of those videos, consisted of *r* = 0.74. Disagreements about the coding were discussed to reach a consensus.

#### Questionnaires

Dimensions of Discipline Inventory (DDI; Straus and Fauchier, 2007): the DDI measures parents’ opinions about doing the 26 most frequently used discipline behaviors, such as explaining, rewarding, deprivation of privileges, and spanking. Items are grouped into nine sub scales and two global scales: punitive discipline (Corporal Punishment; Deprivation of Privileges; Penalty Tasks and Restorative Behavior; and Psychological Aggression) and non-punitive discipline (Diversion; Explain/Teach; Ignore Misbehavior; Monitoring; and Reward). The response scale ranges from *1 = never* to *4 = (almost) always*. The average item score was computed for both punitive and non-punitive discipline sub-scales with Cronbach’s values of α = 0.85 and α = 0.78, respectively.

Perceived Environment Index (PEI; Pluess et al., 2017)^2^: The PEI derives from the PREI questionnaire (Perceived Refugees Environment Index) originally used with refugees (see text footnote 2). All questions are appropriate to detect individual perceptions of the quality of the environment in socioeconomically disadvantaged populations. We selected 27 items divided into six scales: Basic Needs (access to food, water, and clothing; three items), Housing (quality of house condition; seven items), Community Social Environment (support and feeling of safeness in the community; five items), Work (quality of salary and working condition; three items), Learning (quality of children learning and play opportunities; five items), and Family Environment (quality of emotional and practical support received by family members; four items). Caregivers were asked to evaluate their perception on a five-point Likert scale (*1 = Not at all, 5 = Yes*). Cronbach’s *α* values were 0.64, 0.79, 0.71, 0.66, 0.70, and 0.75, respectively.

## Results

### Aim 1: assessing sensitivity, level, and range of sensitivity scores

The feasibility of assessing sensitivity with the Ainsworth scale was confirmed by the reliability between coders, *r* = 0.74, high for observational data, which indicated that the manual gives enough information to apply the coding system to this population. Moreover, all the caregivers and children consented to being filmed and seemed comfortable in front of the camera confirming the possibility of video recording spontaneous caregiver–child interaction to assess caregiver sensitivity.

**Figure 1:**
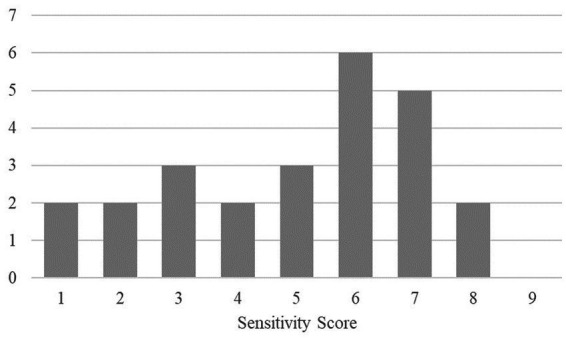
Distribution of sensitivity scores (from 1 to 9) in the Sample.

Addressing the second part of the first research aim, we examined the sample’s level and range of sensitivity scores. The Ainsworth sensitivity scores ranged from 1—highly insensitive to 8—very sensitive, with an average presence of marginal sensitivity (*M* = 5.00; *SD* = 2.12). Figure 1 shows the distribution of the sensitivity scores. This distribution showed that the scale is applicable to this sample, as it captured the whole range of possible sensitivity levels of caregiving.

### Aim 2: descriptions of manifestations of sensitivity

Descriptions of the (in)sensitive interactions in this cultural context are provided to address the second research aim. Different from caregiver–child observations previously conducted in Western countries that mainly consisted of play with toys interactions, in this sample, caregivers’ activities varied (and occurred in combination within families). A total of 15 caregivers engaged in feeding (preparing food or coffee, giving food to the child, and breastfeeding), 14 played with the child (playing with toys, singing a song, dancing, drawing, and chatting), eight engaged in body care (washing hair or the whole body, brushing the hair, and dressing the child), and three choose housekeeping (washing clothes or dishes and cleaning the house). The distribution of activities was quite similar in the caregivers with low and high sensitivity scores. To help researchers and clinicians assess sensitivity in similar populations, our findings are organized in paragraphs corresponding to each of the main activities in which the caregiver and the child were observed to engage with descriptions of sensitive and insensitive caregiver behaviors.

#### Feeding activities

Activities related to food (15 dyads) consisted of young children breastfeeding (two dyads) and older children mainly making traditional coffee together. In Ethiopian culture, drinking coffee together is a unique traditional moment and consists of a specific order of tasks. By asking for help in preparing coffee, the caregiver also teaches the child how to accomplish this special tradition. Sensitive caregivers used this activity as a chance to share dyadic positive interactive moments with their children. The following interaction is an example.

*The caregiver and the child are on the sofa. The caregiver is holding the child on her knees. The child looks at the camera and does not focus on the food. The caregiver proposes to do a game: the child gives food to the caregiver and the caregiver offers food to her. The child smiles and starts playing and eating. Before giving the child a new piece of food, the caregiver waits for her to finish the last piece and for her turn to play. They smile and talk for several minutes. (3-year-old girl).*

The caregiver refocused the child’s attention on the food by making it look like a game in which the role of the child is not passive but active.

*The caregiver takes the bottle and tries to get the child to drink. The child turns her head away and extends her hand open to the caregiver. The caregiver puts a drop of water on the child’s hand, and she smiles. The child smiles. They are both silent. (24-month-old girl).*

When an activity was not appreciated or understood by the child, the sensitive caregiver understood it and she stopped and adapted her behavior according to the child’s needs. Even if putting water on the hand was not a conventional action, this caregiver understood and accepted the child’s idea and followed it, transforming the drinking into a playful moment. This happened without the need to use words, either for the child to communicate her needs or for the caregiver to respond appropriately. Chatting and talking are not always necessary to be sensitive.

The following example is from a caregiver who received a score of 1.

*The caregiver feeds the infant with the baby bottle. She puts the bottle in the baby’s mouth, but the baby does not suck and looks away. The caregiver keeps trying and after a while she forces some milk into the baby’s closed mouth. The baby spits. (5-month-old boy).*

The baby’s negative feedback to the caregiver’s activity was refusing to suck the bottle. The low-sensitive caregiver did not understand the signal and kept being concentrated only on feeding as a task. This passive way of reacting to activities, which the child did not like, was very frequent in this sample. The children rarely cried and rarely used negative vocalizations to show their discomfort.

#### Play activities

Even though toys were almost never present in the houses of these families, a considerable number of caregivers chose to play with their children (14 dyads). We defined play as each playful activity, without a precise aim other than enjoying the time together, including dancing and singing together, drawing, chatting without doing anything else, and playing with objects.

*The child repeats many times the word “ma-ma” and other sounds. The caregiver repeats the sounds made by the child. She tries to complete the words, looking at the child’s eyes to check if she got them correct. The child smiles. (19-month-old boy).*

This was an example of the caregiver reading and understanding the meaning of even the child’s subtle and minimal communicative cues. The sensitive caregiver also waited to check if she got the correct sense of the child’s signal. This is particularly necessary for young children with limited vocabulary size and whose communicative signals are often not easy to understand.

The play activities were also helpful in exploring how caregivers dealt with children’s adverse reactions and non-compliant behaviors.

*The child looks bored; he takes the caregiver’s hand and tries to bring her outside. The caregiver says “Do you want to go? Yes, we will go,” she smiles and then she gently touches his shoulder, directing him back into the room, and she starts speaking with the child describing a colorful box in the room. The child goes back inside the room. (19-month-old boy).*

The sensitive caregiver noticed and understood the child’s signal of discomfort. Caregivers in this sample very rarely labeled emotions. More often they chose, as this caregiver did, a distraction strategy by focusing the child’s attention on something else that was appropriate to the child’s interests.

On the other hand, low-sensitive caregivers found playing with their children difficult.

*The girl is playing with a small chair. The caregiver takes a teddy bear and puts it close to the child’s face. The child looks scared, runs away, and goes to play with teacups. The caregiver puts the teddy bear in front of her on the table. The child keeps playing with teacups. (14-month-old girl).*

This caregiver tried to dictate play and could not follow the child’s interests or focus of attention. The way she tried to play was intrusive, and she did not recognize the child’s fear when she first saw the teddy bear.

#### Body care

Caregivers who chose to do body care activities mainly did it outside the house using water from a basin because the bathroom and running water were not present in the houses. Hair brushing consisted of putting oil on the child’s head to soften the hair and brushing the hair. Sensitive caregivers used these occasions to share affect and positive emotions with the child, making the body care experience a relaxed and positive routine activity. Below are examples of such situations.

*The caregiver is brushing the child’s hair and changing his clothes. Before each movement, she explains to the child what she will do; she smiles, and the child is held gently and receives many caresses. When brushing the child’s hair, the caregiver asks the child questions about his nursery day. (19-month-old boy).*

*When brushing the child’s hair, the child often scratches her hair and the caregiver promptly puts on more oil and does a light massage where the child indicates it itches. (5-year-old girl).*

This second example includes both verbal and non-verbal positive responses showing that the presence of sensitivity was not strictly linked with chatting and verbal comments.

Low-sensitive caregivers did the body care routine more as a chore and focused more on accomplishing the activity than on using it as a dyadic interactive moment. Interactions lacked verbal or non-verbal appropriate responses. Low-sensitive caregivers often positioned themselves behind the child, not facilitating face-to-face interaction with the child, and hampering the observation of the child’s needs. The following dyadic moment is an example.

*The caregiver is washing the child’s hair by putting water on her head while her head is upside down. The child silently keeps touching her eyes because the soap gets inside them, but the caregiver keeps putting water on the child’s head. When finished, the caregiver puts cream on the child’s face. When the cream gets in her eyes, she tries to clean them with the sleeve of her dress. (33-month-old girl).*

This child, even if young, sat and let the task happen without adverse reactions or explicit complaints.

##### Housekeeping activities

A total of three caregivers chose to do housekeeping asking their children for help to wash clothes and the floor. Two were caregivers of girls and one of a boy, the ages varied from 3 to 8 years old. Sensitive caregivers used these interactive moments as an occasion to chat and share a pleasant dyadic moment to teach the child a housekeeping activity.

*The caregiver and the child are washing clothes. Each is washing a piece of clothing separately, for the first half of the interaction. After a while, the caregiver takes a recipient and fills it with water. The child looks at her with an interested look. The caregiver gives him the recipient and allows him to pour water on the clothes while she holds them. They go on like this for the rest of the interaction, washing together and talking. (8-year-old boy).*

This caregiver understood and appropriately responded to the child’s signals, adapting the task to the child’s needs and desires. This behavior caused a significant change in the interaction, they moved from a parallel activity (both washing separately) to a dyadic activity (each of them had a role in the interaction and cooperated).

Low-sensitive caregivers usually kept the focus on accomplishing the housekeeping task rather than on the dyadic experience, as the following one.

*The caregiver and the child are cleaning the floor. The older sister sits on the sofa. The child keeps looking at the sister. The child cleans hastily keeps looking at the sister and not paying attention to the caregiver when the caregiver tells her what to do. The caregiver does not acknowledge the child’s interest in her sister. She keeps telling the child what to do and cleans the floor of the other part of the room. They never look at each other. (8-year-old girl).*

This child did not seem to enjoy the activity. The caregiver focused on the task and kept giving her orders without paying attention to the child’s needs and desires.

### Aim 3: association between sensitivity, caregiver discipline behaviors, and perceived quality of the environment

To address the third research aim, we examined the associations between caregiver sensitivity and socioeconomic family characteristics (child gender and age, number of family members, and caregiver education), caregivers’ reports of discipline behaviors (DDI punitive and non-punitive discipline scores), and perception of the quality of the environment (PEI scale scores). To assess patterns of factors that cluster with sensitivity in a way that is less hampered by the small sample size, a k-means cluster analysis was conducted (see Table 2). This procedure employs an algorithm that identifies relatively homogeneous groups of cases based on selected characteristics and it provides information about patterns rather than a fully predictive model. Continuous socioeconomic variables were dichotomized as follows: child aged older than 4 years, number of family members above four living in the same house, and caregiver education above 5 years of school attended. Continuous variables (questionnaire scores) were coded as follows: *0 = below the median* and *1 = equal or above the median*. The analysis revealed that compared to the nine caregivers with low sensitivity scores (mean sensitivity score = 3), the 16 mothers with high sensitivity scores (mean sensitivity score = 6) were characterized by more satisfaction with their housing and family environment condition. Socioeconomic variables did not distinguish between the lower and higher sensitivity groups. Moreover, non-punitive or punitive discipline did not distinguish significantly between the two groups but did show patterns consistent with expectations (i.e., higher sensitivity clustered with less punitive discipline).

**Table 2:** Cluster analyses.

	Cluster 1 (*n* = 16)	Cluster 2 (*n* = 9)	*F*	*p*
Sensitivity Score	6	3	80.6	<0.01
Gender (Male)	31%	33%	0.01	0.92
Child Age > 4 years	56%	33%	1.18	0.29
N. of family members >4	81%	44%	3.85	0.06
Caregiver education >5 years	69%	33%	3.06	0.09
DDI Punitive Discipline	31%	56%	1.17	0.29
DDI Non-Punitive Discipline	63%	33%	1.96	0.17
PEI Basic Needs	63%	33%	1.96	0.17
PEI Housing	75%	33%	4.60	0.04
PEI Community Social Environment	69%	44%	1.38	0.25
PEI Working	44%	67%	1.17	0.29
PEI Learning	56%	56%	0.01	0.97
PEI Family Environment	81%	33%	6.85	0.01

Because all the variables were coded as 0 = no 1 = yes, the averages of those variables in each cluster reflect percentages. For easier interpretation, these averages are reported as percentages. Continuous variables were coded as follows: 0 = below the median, 1 = equal or above the median. DDI = Dimensions of Discipline Inventory. PEI = Perceived Environment Index.

## Discussion

To our knowledge, this is the first study reporting on sensitivity in Ethiopia using observations of spontaneous caregiver–child interactions recorded at home. The first significant contribution of the study (Aim 1) is showing that assessing caregiver sensitivity using video recordings of caregiver–child interactions is possible and not even that difficult to realize in this population. Caregivers easily accepted being filmed, generally showed very few signs of camera shyness, and just went about their business without minding the camera. This was also possible because the researchers were introduced by people to the families felt familiar with. Within this not-intrusive situation, inside their housing environment, caregivers felt free to choose the kind of activity they preferred and engaged with the child in various and specific ways. This includes this study within the recent field aiming to observe sensitivity in non-western populations and suggests the need and possibility to go further with it (Mesman et al., 2018a; Mesman, 2020b).

The usefulness of the traditional Ainsworth’s sensitivity scale (Ainsworth et al., 1974) for coding sensitivity in this context was illustrated by the fact that almost the full range of scores was observed, with a high agreement between observers. Moreover, we did not encounter situations or videos that we could not code with the scale, a further demonstration that formulations of the scale are applicable to this cultural context as well. More than half of the caregivers were found to be at least more sensitive than not sensitive. This showed that despite their harsh economic conditions, difficult living circumstances, lack of money, and learning opportunities, many caregivers responded appropriately to their children’s needs. Sensitivity was not categorically absent but showed clear variation from low to high even in such deprived circumstances, consistent with previous studies conducted in similar contexts (Mesman et al., 2016a, 2018b).

From the observations of caregiver–child interactions, we extracted the descriptions of typical activities conducted in sensitive and insensitive ways according to the attunement or not to infant needs (Aim 2). This description can represent a valuable guideline for researchers and clinicians who want to assess sensitivity in this or similar contexts. Consistent with previous studies that examined caregiving practices, the type of activity proposed by caregivers to the child varied and some differed from a traditional Western caregiver–child interaction (Bornstein et al., 2015; Bornstein, 2019). While most Western studies are based on the observation of sensitivity manifestations mainly during play with toys, the present study’s caregivers also engaged the child in different daily activities. However, each activity was manifested in high-sensitive and low-sensitive caregivers, showing that the quality of parenting is not related to the activity chosen but to how this activity is conducted, as previously demonstrated in other cultural contexts (Mesman, 2020b).

Even if toys were rare in these houses, more than half of the caregivers decided to play with the child in many ways, such as chatting, singing, dancing, or drawing. This demonstrates that the absence of toys does not mean the lack of playful moments as an opportunity to share dyadic effect and knowledge. The sensitive play was characterized by following the child’s focus and needs, i.e., by commenting on the focus of interest, answering the child’s questions, and reinforcing the sharing of experiences, as well as by sharing positive affect and responding to the child, even if slight signals of distress. Many caregivers involved their children in typical daily activities in Ethiopia, such as preparing traditional coffee, eating, taking care of hair dreads, and accomplishing housekeeping tasks. These daily routines are essential occasions to transmit cultural traditions and habits while sharing playful dyadic caregiver–child moments (Coe and Clark, 2021). Sensitive caregivers were open to changing the path of the task according to the child’s needs, i.e., playing with water if the child started to do it and letting the child lead the activity.

Maybe the reason why previous studies reported a lack of pleasant sharing child–caregiver moments in Ethiopia is that they were looking for Western typical dyadic moments, such as playing with toys together and reading books, leaving out all the other important dyadic activities that characterize these child–caregiver interactions (Ringness and Gander, 1974; Cherie and Berhanie, 2015; Coe and Clark, 2021). For instance, Ringness and Gander (1974) reported the prevalence of unsupervised play in young Ethiopian children, a behavior not present in American families. However, according to our observations, this does not mean that Ethiopian parents do not share pleasant dyadic moments with their children. Observing the quality of spontaneous daily activities allows for a better understanding of how Ethiopian caregivers and their children share time. Indeed, high school students who reported the lack of fun moments also reported highly positive communication and feeling of closeness between family members (Cherie and Berhanie, 2015) that they may have experienced during daily life routines such as those described in the current study. This aspect should also be taken into consideration during parent–child interventions. Assessing caregiver sensitivity focusing on an interaction that involves playing with toys, as often happens in Western cultures, might not allow the caregiver to fully express her responsive abilities because the activity is far from their usual dyadic experience. Similarly, interventions based on using toys to enhance sensitive caregiver responses are also likely less appropriate for this population. It could even be counterproductive as it might provoke a sense of inadequacy in these parents who cannot afford to provide toys to their children and might suggest that sensitive parenting is only relevant to play. The descriptions of activities and their expressions in sensitive and insensitive ways provided in our study represent a helpful guideline to avoid such problems and facilitate the evaluation and observation of culturally specific behaviors through the lens of sensitivity.

A notable characteristic of the children’s behaviors in this selected group was a limited expression of explicit negative affect such as showing anger or sadness as a response to insensitive caregiver behaviors. Even children with low-sensitive caregivers tend to show very few adverse reactions to express their lack of interest or pleasure in the task. They mostly showed compliance behaviors showing passivity and low engagement in the activity. This is typical of this culture (Tilahun, 2002; Gota, 2012), and it has to be considered when analyzing sensitivity in this population with implications for the structure of interventions. Because one aspect of sensitivity is to be able to notice and adequately respond to a child’s negative signals, care professionals must consider the minimal manifestations of negative affect typical of these children and work to facilitate caregivers’ awareness of these signals.

The cluster analyses (Aim 3) showed that sensitivity does not go together with a particular discipline style. While previous Western studies showed an association between sensitivity and positive discipline (Joosen et al., 2012), sensitive parents might use punitive as well as non-punitive discipline behaviors in this culture. Our findings align with Ethiopian studies reporting the co-occurrence of closeness and unquestioning obedience to parental rules as a typical way to manage discipline in vertical collectivist cultures (Cherie and Berhanie, 2015; Gelan, 2016). A deep exploration of the discipline strategies used in this population, particularly among low-sensitive caregivers would help identify the focus of the intervention when the aim is to promote sensitive discipline.

Sensitivity clustered with high perceived environment satisfaction; the most sensitive caregivers are also those happiest with their living conditions. Because the difficult living conditions of families in our sample were relatively homogeneous, this result stresses the importance of the perception of satisfaction and the feeling of having enough resources to guarantee family life’s basic needs. This perceived satisfaction may reduce the stress that previous studies reported being associated with harsh economic situations and therefore represents an important protective factor for the quality of parenting (Rijlaarsdam et al., 2013; Hoff-Ginsberg and Laursen, 2019; Alsarhi et al., 2020; Rahma Alsarhi et al., 2020). We may expect that an effective intervention oriented to populations with similar economic restraints should not only focus on the parent–child relationship but also on the quality of the housing condition of the family by supporting those families both from psychological and economic perspectives (Bornstein, 2019).

Another perceived environmental factor clustering with sensitivity was the quality of the family environment, which reflects the perception of support and help from people living in the house. This is a predominantly collectivist culture where togetherness, support, and interdependence are considered fundamental values, and people count on community support. A positive family environment is characterized by sharing parenting duties and might constitute an essential source of support for the caregiver, facilitating sensitive parenting. Interventions might focus on helping these families, particularly isolated families, by structuring connections with the new local community to support each other (Shuey and Leventhal, 2019).

Some limitations of the present study should be addressed. First, even if our sample is homogeneous enough, it is also limited, and further explorations with larger samples are necessary to confirm our findings. Moreover, we did not collect children’s outcomes and conclusions about the effects of sensitivity on children’s development in this population cannot be addressed, which also precludes clear conclusions about the validity of the Ainsworth observation instrument in this population. This should be the focus of future studies. Finally, the videos were coded by Western researchers trained in assessing sensitivity (one with extensive experience in coding sensitivity across cultures). Training local researchers and health workers would contribute to a more culturally contextualized view of the phenomenon of sensitivity and more inclusive science. The aim is to add such procedures to future analyses of the data. Concerning the third aim, a larger sample size would allow us to explore the patterns of factors associated with sensitivity with more robust predictive analyses.

Despite the limitations of the study, which narrows the generalization of results, the indications of our study provided about the assessment of sensitivity could be taken into consideration in other similar populations. The socioeconomic condition of the families with its implications, such as the lack of learning and supporting opportunities both for the child and the caregiver, the lack of toys, and reduced living space for children, is very common in sub-urban and urban areas of developing countries. Moreover, some rearing conditions, such as children living with several people, are also typical of collectivistic cultures. To help the generalization of our findings to populations with similar characteristics, we provided some guidelines in Table 3.

**Table 3:** Guidelines for assessing sensitivity in low-income populations.

Video-recording
Conduct a brief familiarization with the researcher and the camera if the child and the caregiver are not used to it. The interaction should be recorded in the home environment (not in a laboratory) letting the caregiver to choose the material to use. Do not explicitly refer to play, let the caregiver choose the activity she/he prefers to do with the child. Do not bring toys from the lab, the caregiver could be not used to play with the child using toys.
Considerations for the assessment of sensitivity
The quality of parenting is not related to the activity chosen but to how this activity is conducted. Play can be seen in many ways (i.e., chatting, singing, dancing, drawing, and playing with objects) not only by playing with toys. Typical daily activities (i.e., preparing food, eating, taking care of hair dreads, and accomplishing housekeeping tasks) can be a useful occasion to observe. Sensitive caregivers use daily activities as a chance to share dyadic positive interactive moments with their children. Compliance behaviors showing passivity and low engagement in the activity are an expression of negative child feedback to the caregiver’s behavior as well as anger or sadness. Look at both verbal and non-verbal caregiver positive responses; chatting and talking are not always necessary to be sensitive. Caregiver reading and understanding the meaning of even the child’s subtle and minimal communicative cues is an indication of sensitivity. The sensitive caregiver also waits to check if she/he got the correct sense of the child’s signal. Low sensitive caregivers often do not adapt their behavior to the child’s signals and keep being concentrated only on the task. Low sensitive caregivers often position themselves behind the child, not facilitating face-to-face interaction with the child.
Considerations for interventions
Check the economic condition of the family and include socio-economic support, when possible, to help promoting sensitivity. Keep in mind the considerations on assessing sensitivity also for the intervention. Do not focus the intervention on the use of toys but on activities the caregiver and the child are used to conducting together. Promote the caregiver’s ability to read subtle and not explicit signals of discomfort. If possible, conduct a home-based intervention to help the caregiver find sensitive ways to interact with the child in their environment.

## Conclusion

This study contributed to understanding culturally specific characteristics and interaction contexts of sensitivity. We found that poor socioeconomic living conditions are not necessarily associated with less adequate parenting. Evident variability in the presence of sensitive caregiving is present across caregiver–child interactions during various daily and culturally-based tasks in the home setting. The non-prescriptive nature of the Ainsworth sensitivity scale (Ainsworth et al., 1974), in terms of specific behaviors, makes it particularly suitable for an open and culturally inclusive approach to understanding sensitivity to children’s signals and needs. This approach should focus on helping local care professionals to be aware of the different activities and ways relevant for recognizing aspects of sensitivity in their community, going beyond the idea of Western-based methods and guidelines that tend to dominate professional education in non-Western regions as a result of (post)colonial mechanisms (Chapin, 2018; Bornstein, 2019). Investing in decolonizing knowledge about early childhood caregiving can enrich preventive and supportive counseling and social interventions for families across the globe.

## Data availability statement

The raw data supporting the conclusions of this article will be made available by the authors, without undue reservation.

## Ethics statement

The studies involving human participants were reviewed and approved by Department Neuroscience, Imaging and Clinical Sciences of the University G. D’Annunzio Chieti-Pescara. Written informed consent to participate in this study was provided by the participants’ legal guardian/next of kin.

## Author contributions

MS and JM: conceptualization, writing—original draft, and formal analysis. EP and MF: conceptualization and writing—review and editing. TK, MA, MG, TT, and AG: data curation and writing —eview and editing. All authors contributed to manuscript revision, read, and approved the submitted version.

## Funding

The present study was supported by Il Sole Onlus (www.ilsole.org).

## Conflict of interest

The authors declare that the research was conducted in the absence of any commercial or financial relationships that could be construed as a potential conflict of interest.

## Publisher’s note

All claims expressed in this article are solely those of the authors and do not necessarily represent those of their affiliated organizations, or those of the publisher, the editors and the reviewers. Any product that may be evaluated in this article, or claim that may be made by its manufacturer, is not guaranteed or endorsed by the publisher.

## Footnotes

^1^IFSO is located in Addis Ababa and designs and implements child and family focused service delivery projects; while Il Sole Onlus is located in Italy and provides financial donation, technical and professional supports.

^2^Pluess, M., Annan, J., Dow, J., Karam, E. G., Tubbs Dolan, C., and Aber, J. L. (2017). The Perceived Refugee Environment Index (PREI). [Unpublished Manuscript].
